# Runx2 Haploinsufficiency Ameliorates the Development of Ossification of the Posterior Longitudinal Ligament

**DOI:** 10.1371/journal.pone.0043372

**Published:** 2012-08-21

**Authors:** Makiko Iwasaki, Jinying Piao, Ayako Kimura, Shingo Sato, Hiroyuki Inose, Hiroki Ochi, Yoshinori Asou, Kenichi Shinomiya, Atsushi Okawa, Shu Takeda

**Affiliations:** 1 Department of Orthopedic Surgery, Tokyo Medical and Dental University, Tokyo, Japan; 2 Hard Tissue Genome Research Center, Tokyo Medical and Dental University, Tokyo, Japan; 3 Section of Nephrology, Endocrinology and Metabolism, Department of Internal Medicine, School of Medicine, Keio University, Tokyo, Japan; The Hebrew University, Israel

## Abstract

Ossification of the Posterior Longitudinal Ligament (OPLL) is a disease that is characterized by the ectopic calcification of the ligament; however, the pathogenesis of OPLL remains to be investigated. We attempted to identify the *in vivo* role of Runx2, a master regulator of osteoblast differentiation and skeletal mineralization, in the pathogenesis of OPLL. The expression of Runx2 in the ligament was examined using *in situ* hybridization and immunohistochemistry and by monitoring the activity of a *LacZ* gene that was inserted into the *Runx2* gene locus. To investigate the functional role of Runx2, we studied *ENPP1^ttw/ttw^* mice, a mouse model of OPLL, that were crossed with heterozygous *Runx2* mice to decrease the expression of Runx2, and we performed histological and quantitative radiological analyses using 3D-micro CT. Runx2 was expressed in the ligament of wild-type mice. The induction of Runx2 expression preceded the development of ectopic calcification in the OPLL-like region of the *ENPP1^ttw/ttw^* mice. Runx2 haploinsufficiency ameliorated the development of ectopic calcification in the *ENPP1^ttw/ttw^* mice. Collectively, this study demonstrated that Runx2 is expressed in an OPLL-like region, and its elevation is a prerequisite for developing the complete OPLL-like phenotype in a mouse model of OPLL.

## Introduction

Ossification of the Posterior Longitudinal Ligament (OPLL; OMIM 602475) is common in East Asia, with a rate of incidence of 2 to 4% [1], [2]. At present, the cause of OPLL remains unclear. Previous reports suggested that OPLL is a multifactorial disease that results from several factors, including a history of trauma, infection, diabetes and HLA antigens [1]. Of note, the predominance of OPLL in a specific ethnic group, such as the Japanese population, suggests that OPLL might arise from hereditary factors [2]. In fact, the incidence of OPLL increases significantly to approximately 30% among family members of second-order relatives of the affected patient and up to 85% in siblings of an affected monozygotic twin [2]. Moreover, there are several reports showing the association of SNPs in several genes and the incidence of OPLL by population-based case-control study. Those include Bone Morphogenetic Protein 4(BMP4) SNPs in Chinese population [3], interleukin 15 receptor alpha(IL15RA) SNPs in Korean patients [4], collagen 6A1(COL6A1) SNPs in Chinese Han population [5] and Transforming Growth Factor-β1 (TGFβ1) SNPs in Japanese patients [6]. However, because multiple genetic and environmental factors are related to the development of OPLL, no causal genetic mutation for the OPLL has been identified [7].

Pathological examinations revealed that the affected lesion in OPLL exhibits characteristics of ectopic bone formation (i.e., the existence of osteoblasts), including a lamellar bone structure that contains well-developed Haversian canals and marrow cavities [8], suggesting that bone formation plays a role in the onset and progression of OPLL.

Runx2 is a master regulator of osteoblastogenesis and, thereby, a regulator of the cells that are responsible for bone formation [9]. Runx2 is essential for the differentiation of osteoblasts from mesenchymal cells, and the forced expression of Runx2 transdifferentiates fibroblasts into osteoblasts. Moreover, *Runx2*−/− mice completely lack osteoblasts, lamellar bone and marrow cavities [10], i.e., the characteristic of affected regions in OPLL, throughout their bodies. However, the pathophysiological role of Runx2 in the development of OPLL has remained unknown.

In this study, we used *ENPP1^ttw/ttw^* mice [11], a mouse model of OPLL, and Runx2 mutant mice to investigate the role of Runx2 in OPLL. We found that Runx2 is induced prior to the formation of ectopic bone in OPLL and that Runx2 haploinsufficiency ameliorates OPLL-associated ectopic calcification.

## Materials and Methods

### Animals

*Enpp1^ttw/ttw^* mice were obtained from the Central Institute for Experimental Animals. *Runx2* ^+/−^ mice have been described previously [10]. We housed all mice under a 12-hr light/dark cycle with *ad libitum* access to standard food and water. We determined the genotypes of the mice by polymerase chain reaction (PCR). (A list of the PCR primer sequences is available upon request.) All animal experiments were performed with the approval of the Animal Study Committee of Tokyo Medical and Dental University (Permit No. 2011-136) and conformed to all relevant guidelines and laws.

### Histological Aalysis

For histological examination, after dissection, the tissue samples were fixed immediately in 4% paraformaldehyde/phosphate-buffered saline, then dehydrated with gradually increasing concentrations of ethanol and embedded in paraffin. After fixation, the tissue samples from the adult mice were decalcified in 20% EDTA for two weeks before being embedded in paraffin. For LacZ staining, the spines from heterozygous *Runx2*- mice were fixed in 0.2% paraformaldehyde at room temperature for 30 minutes, and then stained overnight in X-Gal solution, as previously described [12]. Immunohistochemical staining using antibody against Runx2 was performed using the avidin-biotin-peroxidase complex method with the ABC Rabbit IgG Kit (VECTOR Laboratories), as previously described. The anti-Runx2 antibodies have been described previously [13]. *In situ* hybridization was performed using ^35^S-labeled riboprobes and the standard protocol, as described previously [13].

### Micro-computed Tomography Analysis

We obtained three-dimensional images of the cervical spine using micro-computed tomography (micro-CT, ScanXamte-E090, Comscantecno Co. Ltd., Tokyo, Japan). Each spine was placed in a plastic tube, and images were reconstructed from 750 projections. Ectopic ossification was quantitatively analyzed using bone analysis software (TRI/3D-BON, Ratoc System Engineering Co. Ltd., Tokyo, Japan).

### Statistical Analysis

All data are presented as the mean ± s.d. (n = 8 or more). We performed the statistical analyses using the Student’s *t*-test. Differences were considered statistically significant when P<.05. The results are representative of more than four individual experiments.

## Results

**Figure 1:**
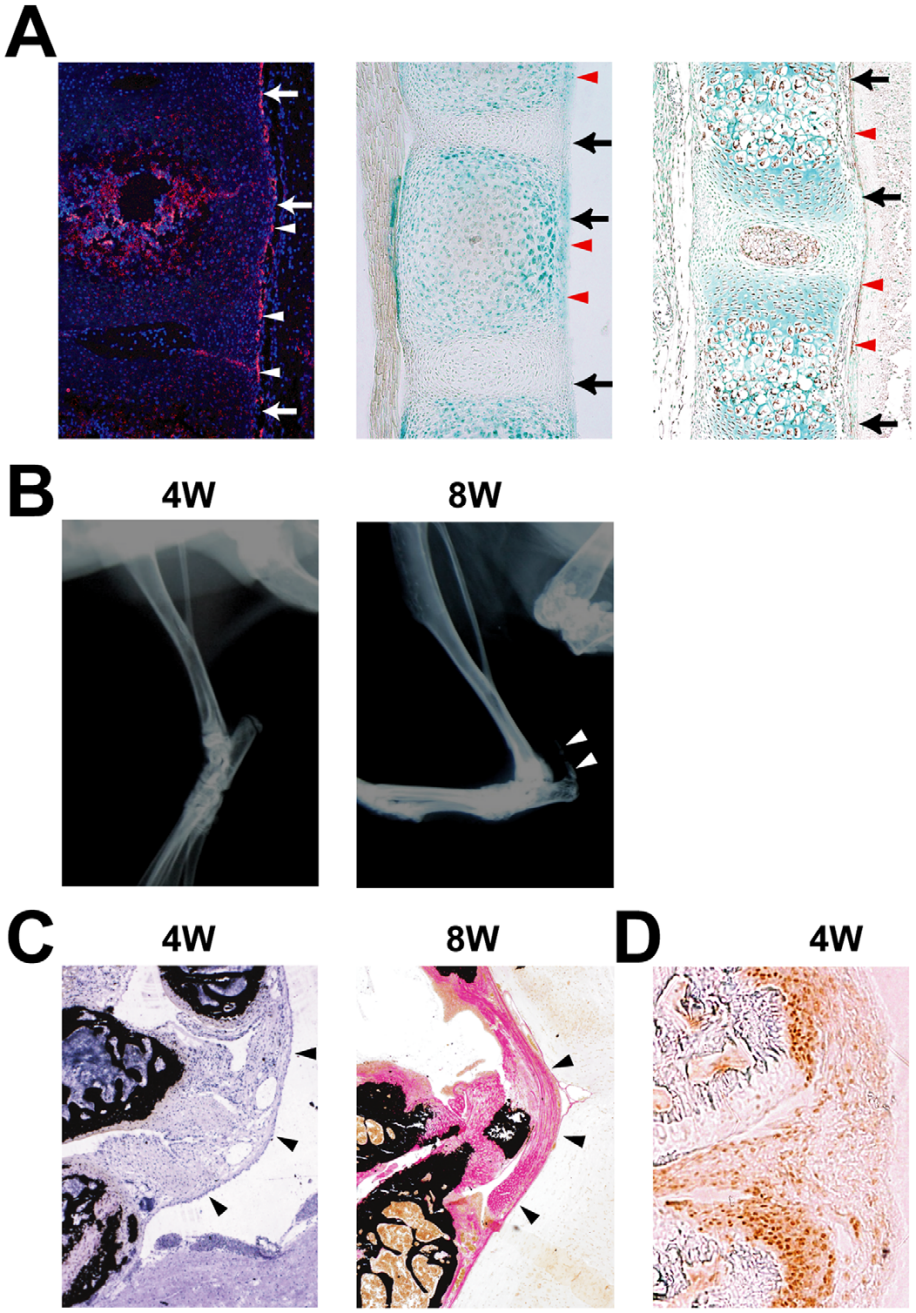
Expression of Runx2 in calcified ligament. A, Runx2 expression (arrowheads) in the posterior longitudinal ligament (arrows). *In situ* hybridization of *Runx2* in wild-type (WT) mouse vertebrae at birth (left). LacZ staining in WT mouse vertebrae at birth (middle). Immunohistochemistry of Runx2 in WT mouse vertebrae at embryonic day 16.5 (right). B, Radiographic assessment of the development of calcification of the ligament in an *Enpp1^ttw/ttw^* mouse at 4 and 8 weeks of age. Note an appearance of calcification at 8weeks (arrowheads) C, Histological assessment of the cruciform ligament (arrowheads) at the atlanto-occipital area in an *Enpp1^ttw/ttw^* mouse at 4 and 8 weeks of age. D, Immunohistochemical staining of Runx2 at the posterior longitudinal ligament in an *Enpp1^ttw/ttw^* mouse at 4 weeks of age. Note that Runx2 was expressed in an area corresponding to the prospective calcification.

As an initial measure of the contribution of Runx2 to ligament development, we analyzed the expression of Runx2 in the prospective ligaments of spine including posterior ligament of the vertebrae at the atlanto-occipital area of mouse embryos using three different experimental techniques. First, we performed an in situ hybridization analysis using Runx2 as a probe. At birth, *Runx2* was expressed in vertebrae and at the edge of the vertebrae, which corresponds to a future ligament (Fig. 1A). Next, we took advantage of the *LacZ* allele that was inserted into the *Runx2* locus [10] by performing LacZ staining of spines isolated from heterozygous Runx2 mice to monitor the expression of Runx2 in developing mouse skeletons at birth. Consistent with our in situ hybridization results, we observed robust expression of Runx2 in both the vertebral body and the adjacent ligament (Fig. 1A). To confirm that Runx2 is expressed in the ligament, we performed immunohistochemistry using an antibody against Runx2 and observed Runx2 protein in the ligament (Fig. 1A). These results demonstrate that Runx2 is expressed in ligament cells.

Next, to address the functional relevance of Runx2 in the development of OPLL, we studied the expression of Runx2 in ectopically calcified lesions, which resemble human OPLL lesions, in *ENPP1^ttw/ttw^* mice, a mouse model of OPLL [11]. *ENPP1^ttw/ttw^* mice are a useful model of ossification in OPLL, which is caused by a point mutation in the *ENPP1* nucleotide pyrophosphatase gene [11]. ENPP1 regulates soft-tissue calcification and bone mineralization by producing inorganic pyrophosphate, a major inhibitor of calcification [11]. *ENPP1^ttw/ttw^* mice did not exhibit any overt abnormalities from birth through four weeks of age. At eight weeks of age, abnormal gait, rigidity of the vertebral column and stiffness of the limb joints developed, as previously reported (Fig. 1B) [11]. A histological examination revealed that an ectopically ossified OPLL-like region developed by eight weeks of age; no calcification was evident at four weeks of age, although proliferation of the ligament cells was noted at that age (Fig. 1C). Because Runx2 is essential for bone formation and mineralization, we tested whether Runx2 expression was observed at four weeks of age in the proliferating cells of the ligament that were subsequently mineralized. In fact, Runx2 expression was clearly observed in the ligament at four weeks (Fig. 1D), the age at which the ligament was not calcified (Fig. 1C). Thus, Runx2 expression precedes the development of an OPLL-like region in the ligament, suggesting that Runx2 may play a role in the development of OPLL.

**Figure 2:**
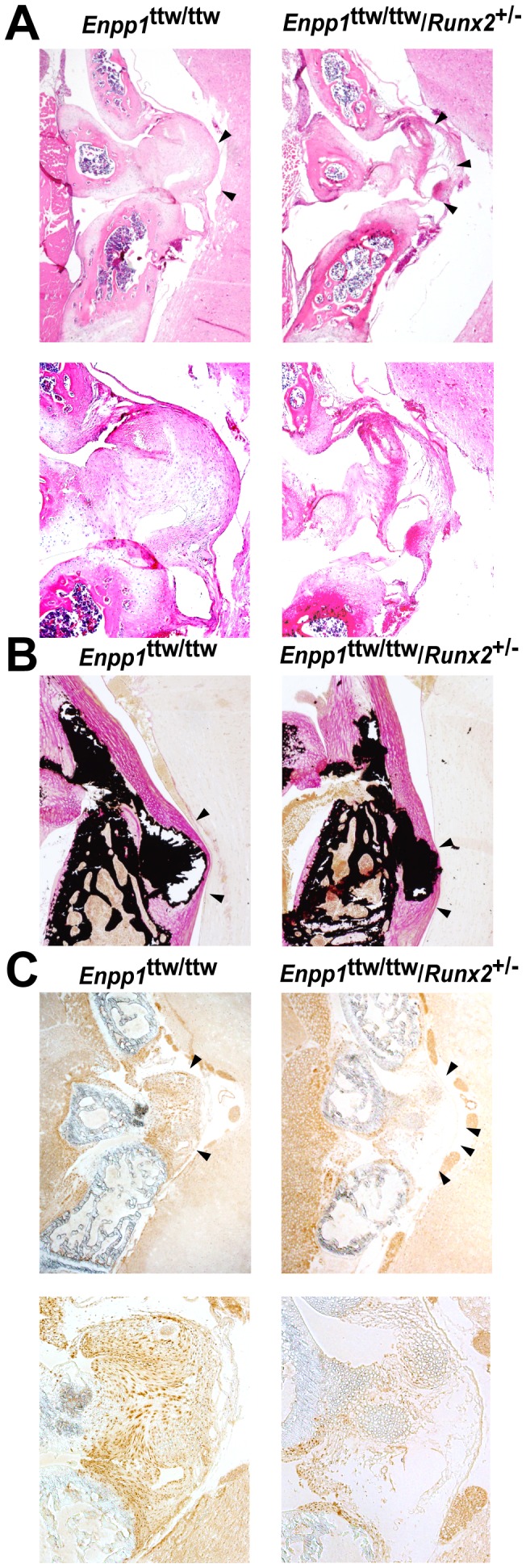
Runx2 haploinsufficiency ameliorates the development of OPLL. A–C, Histological (A and B) and immunohistochemical (C) analyses of the cruciform ligament at the atlanto-occipital area in *Enpp1^ttw/ttw^* mice at 8 weeks (A and B) or 4 weeks (C) of age with (right) or without (left) Runx2 haploinsufficiency. (A: H&E staining; B: von Kossa staining.) Note a decrease in calcified region (B) and Runx2 immunoreactivity (C) in *ENPP1^ttw/ttw^*/*Runx2^+/−^* mice. Bottom panels are higher magnification images.(A and C).

**Figure 3:**
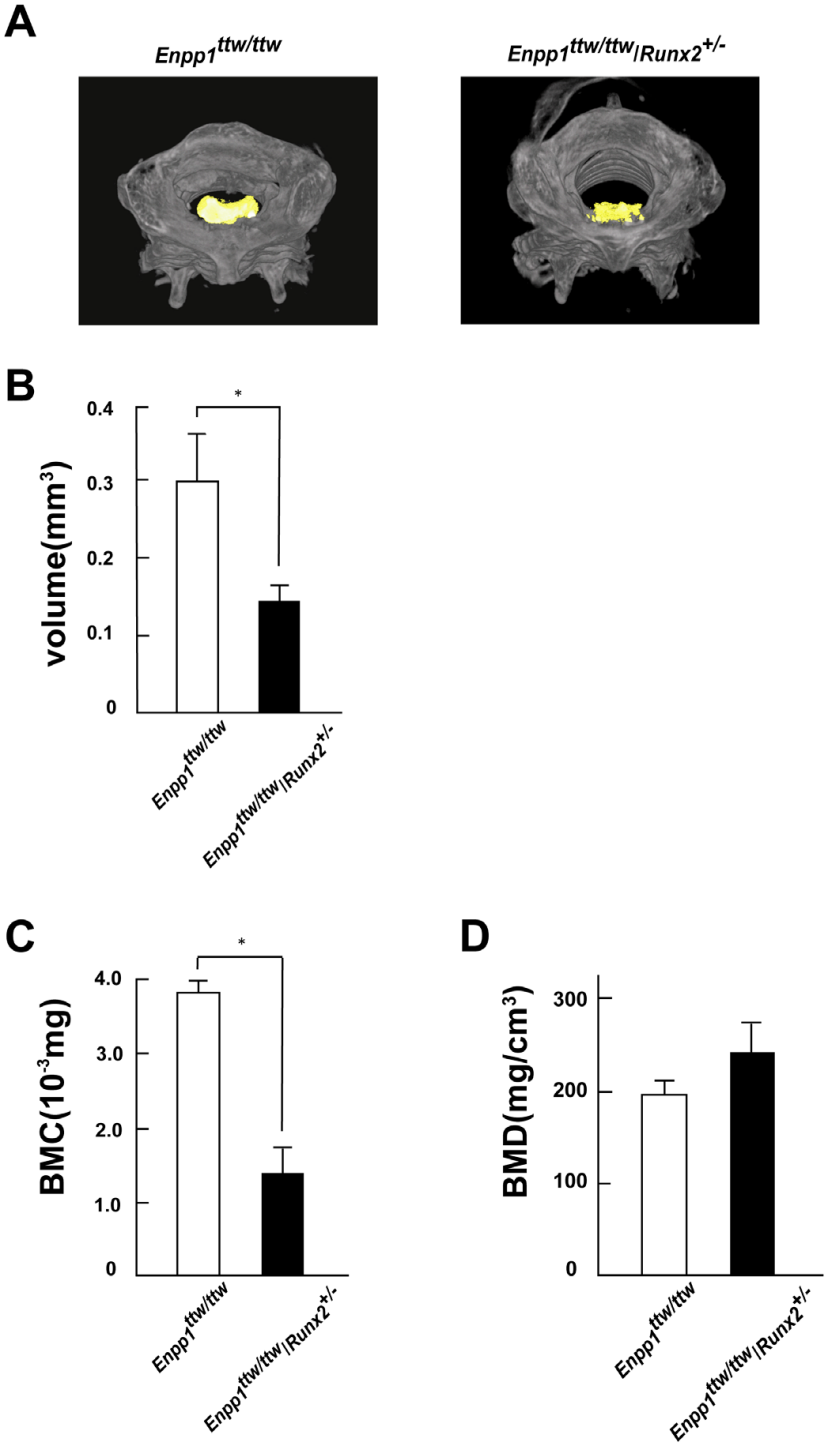
Quantitative analysis of ossification by micro-computed tomography (CT). A, Micro-computed tomography of the cervical spine of *Enpp1^ttw/ttw^* mice at 12 weeks of age with or without Runx2 haploinsufficiency. 3D reconstructed images.Ectopically calcified region is shown in yellow. B, Quantitative analysis of the ossification of the cruciform ligament at the atlanto-occipital area using micro-CT in *Enpp1^ttw/ttw^* mice at 8 weeks of age with or without Runx2 haploinsufficiency. Note a significant decrease in calcified region in *ENPP1^ttw/ttw^*/*Runx2^+/−^* mice. C,D Micro-CT analysis of the ossification of the cruciform ligament at the atlanto-occipital area in *Enpp1^ttw/ttw^* mice at 12 weeks of age with or without Runx2 haploinsufficiency. Bone mineral content (C) Bone mineral density (D).

Next, to address the functional role of Runx2 in OPLL, we tested whether decreasing Runx2 expression affects the development of the OPLL-like region in the *ENPP1^ttw/ttw^* mice. Accordingly, we generated *ENPP1^ttw/ttw^* mice carrying a single allele of Runx2 (*ENPP1^ttw/ttw^*/*Runx2^+/−^* mice) by mating *ENPP1^ttw/ttw^* mice with heterozygous Runx2 mice. A histological examination revealed that the abnormal proliferation of cells in the posterior longitudinal ligament region was substantially lower in the *ENPP1^ttw/ttw^*/*Runx2^+/−^* mice than in the *ENPP1^ttw/ttw^* mice (Fig. 2A). Moreover, the ectopically calcified OPLL-like region was significantly smaller in the *ENPP1^ttw/ttw^*/*Runx2^+/−^* mice (Fig. 2B). An immunohistochemical analysis confirmed that Runx2 expression was lower in the *ENPP1^ttw/ttw^*/*Runx2^+/−^* mice than in the *ENPP1^ttw/ttw^* mice (Fig. 2C). To test rigorously whether Runx2 haploinsufficiency affects the disease progression of OPLL, we quantified the ectopically ossified region in the OPLL model using reconstructed 3D images obtained using micro-CT (Fig. 3A). We noted that all of the *ENPP1^ttw/ttw^* mice exhibited ectopic ossification of the cruciform ligament at the atlanto-occipital area by eight weeks of age (Fig. 2B). Therefore, we quantified the volume of the calcified cruciform ligament. In fact, the volume of calcified ligament in the *ENPP1^ttw/ttw^*/*Runx2^+/−^* mice was less than half of that in the *ENPP1^ttw/ttw^* mice (Fig. 3B). In accordance with that observation, Bone Mineral Content (BMC) of calcified ligament in the *ENPP1^ttw/ttw^*/*Runx2^+/−^* mice was significantly decreased compared to that in the *ENPP1^ttw/ttw^* mice (Fig. 3C). Interestingly, volumetric Bone Mineral Density (vBMD) was not significantly different between the *ENPP1^ttw/ttw^*/*Runx2^+/−^* mice and the *ENPP1^ttw/ttw^* mice(Fig. 3D), indicating that only the areasize of ectopic bone formation was decreased in *ENPP1^ttw/ttw^*/*Runx2^+/−^* mice, while mineral apposition to extracellular matrices per unit volume was not overtly changed. Collectively, these results clearly demonstrate that the removal of one allele of Runx2, which led to a decrease in Runx2 expression, ameliorated the progression of the OPLL-like region that was observed in the *ENPP1^ttw/ttw^* mice.

## Discussion

In this manuscript, we show that Runx2 is expressed in the prospective ligament in mice. We also demonstrate that Runx2 expression is induced in the ectopically ossified area in *ENPP1^ttw/ttw^* mice prior to the appearance of a calcified OPLL-like region. Finally, we demonstrate that decreasing Runx2 expression ameliorates the progress of the OPLL-like region. Although OPLL is characterized by ectopic ossification of the posterior longitudinal ligament, the molecular pathogenesis underlying this ossification in vivo was not known [1]. In this study, we demonstrate for the first time that normal Runx2 expression is necessary to achieve the full development of an OPLL-like region in *ENPP1^ttw/ttw^* mice.

We previously reported that mechanical loading specifically induces Runx2 within the Runx family and that Runx haploinsufficiency ameliorates the intervertebral disc degeneration that is caused by mechanical loading [14]. Interestingly, it is well known that an increase in mechanical loading accelerates the progression of OPLL in human patients [2]. Moreover, previous studies reported that mechanical stress induces Runx2 expression in spinal ligament cells isolated from OPLL patients [15]. Thus, Runx2 induction by mechanical loading may be a common cause of skeletal degeneration, including ectopic ossification and disk degeneration.

We also previously reported that the continuous expression of Runx2 in chondrocytes by the α1(II) collagen promoter-driven *Runx2* transgene led to ectopic bone formation in permanent cartilage (where bone formation is not observed normally) [12]. However, we failed to detect worsening of the OPLL-like region in the *ENPP1^ttw/ttw^* mice using the α1(II) collagen promoter-driven *Runx2* transgene (Iwasaki and Takeda, unpublished observation). This observation can be explained by the fact that the ectopically ossified area in the *ENPP1^ttw/ttw^* mice was not caused by endochondral bone formation [12]. However, given that abnormal chondrocyte proliferation occurs in the affected ligaments of human OPLL patients [1], the putative induction of Runx2 in these chondrocytes may accelerate the progression of OPLL in human OPLL patients.

Interestingly, a recent report suggested that various SNPs in the *Runx2* gene may be associated with an elevated incidence of OPLL in the Han population via an unidentified mechanism [16]. A detailed molecular analysis to investigate whether these SNPs affect Runx2 function is needed. It is also demonstrated that Runx2 expression is enhanced in cells isolated from spinal ligaments in OPLL patients compared to non-OPLL patients [17], [18], however, it remains unknown if altered Runx2 expression is the cause or the result of ossification of the ligament.

Although Runx2 expression was observed in ligaments of wild-type mice at birth, wild-type mice do not usually develop ectopic calcification, as is observed in *ENPP1^ttw/ttw^* mice. Thus, it is possible that Runx2 induction is not sufficient for the development of an OPLL-like phenotype. More importantly, molecule(s) that prevent ectopic calcification–notably, ENPP1–should exist in the ligament of wild-type mice, and these factors may be absent in human OPLL patients. Additional studies are needed to identify these molecules.

Currently, the molecular mechanism underlying the induction of Runx2 in the calcification of prospective ligaments is not known. To date, multiple pathways (e.g., Wnt/LRP5/β-catenin and BMP/Smads) and transcription factors (e.g., MSX2, DLX5, and twist) that regulate the expression and function of Runx2 have been identified [9]. Moreover, promyelotic leukemia zinc finger, a transcription factor which is an upstream regulator of Runx2 and promotes osteoblastic differentiation, is highly expressed in cells isolated from OPLL patients [19].

Therefore, it will be interesting to investigate whether the expression of these machineries that regulate Runx2 expression is altered in *ENPP1^ttw/ttw^* mice and/or human OPLL patients. In fact, several studies have reported a decrease in MSX2 expression [20] or an increase in BMP2 expression [21] in the affected areas of OPLL patients.

To date, no specific treatment for OPLL has been developed. Inhibiting ectopic calcification by substances modulating mineralization such as pyrophosphate is expected to affect systemic bone mineralization [22]. In this paper, we showed that removing one allele of *Runx2* does not affect the degree of mineralization per unit volume, but only affects the areasize of ectopic bone formation. Thus, it is plausible that decreasing ectopic bone formation by inhibition of Runx2 expression may provide novel therapeutic approaches for treating OPLL without affecting mineralization in surrounding normal bone.
